# Cough Frequency During Treatment Associated With Baseline Cavitary Volume and Proximity to the Airway in Pulmonary TB

**DOI:** 10.1016/j.chest.2018.03.006

**Published:** 2018-03-17

**Authors:** Alvaro Proaño, David P. Bui, José W. López, Nancy M. Vu, Marjory A. Bravard, Gwenyth O. Lee, Brian H. Tracey, Ziyue Xu, Germán Comina, Eduardo Ticona, Daniel J. Mollura, Jon S. Friedland, David A.J. Moore, Carlton A. Evans, Philip Caligiuri, Robert H. Gilman, Tuberculosis Working Group in Peru*, Lilia Cabrera, Marco Varela, Francisco Vigil-Romani, Jesus Chacaltana, José L. Cabrera, Antonio Salas, Felix Llanos, Marcos Ñavincopa, Daniela E. Kirwan, Sumona Datta, Jessica D. Rothstein, Nicole A. Doria, Gustavo Hérnandez-Córdova, Richard Oberhelman, Jorge Coronel, Luz Caviedes, Mirko Zimic, Eyal Oren

**Affiliations:** a Laboratorio de Investigación en Enfermedades Infecciosas, Laboratorio de Investigación y Desarrollo, Facultad de Ciencias y Filosofía, Universidad Peruana Cayetano Heredia, Lima, Peru; b Innovation for Health and Development, Laboratory of Research and Development, Universidad Peruana Cayetano Heredia, Lima, Peru; c Laboratorio de Bioinformática y Biología Molecular, Facultad de Ciencias y Filosofía, Universidad Peruana Cayetano Heredia, Lima, Peru; d Instituto Nacional de Salud del Niño San Borja, Lima, Peru; e Asociación Benéfica PRISMA, Lima, Peru; f Escuela Profesional de Ingeniería Física, Facultad de Ciencias, Universidad Nacional de Ingeniería, Lima, Peru; g Facultad de Medicina, Universidad Nacional Mayor de San Marcos, Lima, Peru; h Servicio de Enfermedades Infecciosas y Tropicales, Hospital Nacional Dos de Mayo, Lima, Peru; i Department of Epidemiology and Biostatistics, Mel and Enid Zuckerman College of Public Health, University of Arizona, Tucson, AZ; j Department of Internal Medicine, Cleveland Clinic, Cleveland, OH; k Department of General Internal Medicine, Massachusetts General Hospital, Boston, MA; l Department of Global Community Health and Behavioral Sciences, Tulane University, New Orleans, LA; m Department of Electrical and Computer Engineering, Tufts University, Medford, MA; n Center for Infectious Disease Imaging, Radiology and Imaging Sciences, National Institutes of Health, Bethesda, MD; o Section of Infectious Diseases & Immunity and Wellcome Trust Imperial College Centre for Global Health Research, Imperial College London, London, England; p TB Centre, London School of Hygiene and Tropical Medicine, London, England; q Department of Radiology & Imaging Sciences, University of Utah School of Medicine, Salt Lake City, UT; r Program in Global Disease Epidemiology and Control, Department of International Health, Bloomberg School of Public Health, Johns Hopkins University, Baltimore, MD

**Keywords:** cough, CT, mycobacteria, tuberculosis, CayeCoM, Cayetano Cough Monitor, CXR, chest radiography, HR, hazard ratio, MD, mean difference, MODS, microscopic observation drug susceptibility, RR, rate ratio, TTP, time to positivity

## Abstract

**Background:**

Cough frequency, and its duration, is a biomarker that can be used in low-resource settings without the need of laboratory culture and has been associated with transmission and treatment response. Radiologic characteristics associated with increased cough frequency may be important in understanding transmission. The relationship between cough frequency and cavitary lung disease has not been studied.

**Methods:**

We analyzed data in 41 adults who were HIV negative and had culture-confirmed, drug-susceptible pulmonary TB throughout treatment. Cough recordings were based on the Cayetano Cough Monitor, and sputum samples were evaluated using microscopic observation drug susceptibility broth culture; among culture-positive samples, bacillary burden was assessed by means of time to positivity. CT scans were analyzed by a US-board-certified radiologist and a computer-automated algorithm. The algorithm evaluated cavity volume and cavitary proximity to the airway. CT scans were obtained within 1 month of treatment initiation. We compared small cavities (≤ 7 mL) and large cavities (> 7 mL) and cavities located closer to (≤ 10 mm) and farther from (> 10 mm) the airway to cough frequency and cough cessation until treatment day 60.

**Results:**

Cough frequency during treatment was twofold higher in participants with large cavity volumes (rate ratio [RR], 1.98; *P* = .01) and cavities located closer to the airway (RR, 2.44; *P* = .001). Comparably, cough ceased three times faster in participants with smaller cavities (adjusted hazard ratio [HR], 2.89; *P* = .06) and those farther from the airway (adjusted HR, 3.61;, *P* = .02). Similar results were found for bacillary burden and culture conversion during treatment.

**Conclusions:**

Cough frequency during treatment is greater and lasts longer in patients with larger cavities, especially those closer to the airway.

During 2016, there were estimated to be 10.4 million new TB cases worldwide, causing 1.7 million deaths.^1^ TB is transmitted mostly through coughing,^2^, ^3^, ^4^ which has been associated with increased bacillary burden.^5^ Cough can be assessed easily throughout treatment, but its relationship with cavitary lung disease, to our knowledge, has not been studied.^6^ Identifying radiologic characteristics associated with increased cough frequency is important in understanding transmission and evaluating treatment response.^2^, ^5^, ^7^, ^8^ Infectivity of TB is different for each individual, with some infecting more than others, so transmission in TB is considered heterogeneous.^9^, ^10^, ^11^, ^12^, ^13^, ^14^, ^15^, ^16^, ^17^, ^18^, ^19^ In this longitudinal study, we sought to investigate whether there is an association between cough frequency, and its duration, with radiologic characteristics, such as cavitary volume and cavitary proximity to the airway. We also evaluated whether bacillary burden and culture conversion were associated with these radiologic characteristics.

## Materials and Methods

### Study Design

**Figure 1:**
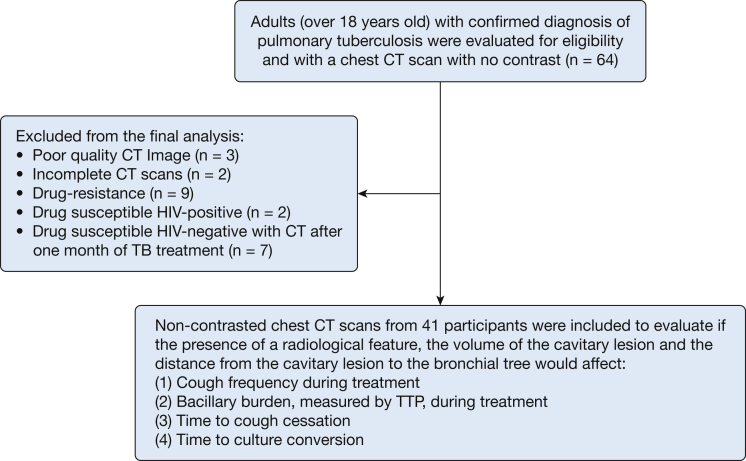
Flowchart for the Cayetano Cough Monitor CT scanning study. Radiologic features are based on readings from a US-board-certified radiologist. Cavity volume and distance to the airway are based on results from a computer-automated algorithm. TTP = time to positivity.

This was a prospective cohort study conducted in two tertiary hospitals in Lima, Peru. The detailed study protocol has been published previously.^20^ Study participants were at least 18 years old, and their pulmonary TB diagnosis and drug sensitivity were assessed by means of microscopic observation drug susceptibility (MODS) broth culture assay.^20^, ^21^, ^22^, ^23^, ^24^, ^25^, ^26^ In this report, we restricted analyses to participants who had a strain that was susceptible to isoniazid and rifampicin and who did not have HIV (Fig 1) because immune status and drug-resistant strains affect radiologic manifestation.^27^, ^28^

The Cayetano Cough Monitor (CayeCoM) was used to record participants’ data daily during the first 14 days of treatment and at days 21, 30, and 60. Recordings started at 9:00 am.^20^ A cough episode included all independent cough events that occurred without a 2-second pause, no cough was a cough frequency ≤ 0.7 cough events per hour, and cough cessation was two consecutive recordings with no cough.^5^ Sputum was obtained on days 0, 3, 7, 14, 21, and 60 of treatment. Bacillary burden was assessed through time to positivity (TTP) of cultures^5^, ^29^, ^30^ in all MODS culture-positive sputum samples, and culture conversion was defined as the first negative culture with no subsequent positive cultures.^5^ The study data for cough frequency and bacillary burden has been published.^5^, ^31^ A baseline chest CT scan was obtained within 31 days of treatment initiation in all participants enrolled in the study who consented, similar to methods used in a previous TB study in participants who were drug susceptible and HIV negative.^28^

### Radiologic Imaging

Scans were obtained (Aquilion 64, Toshiba) and analyzed by using a free Digital Imaging and Communications in Medicine viewer. Our computer-automated algorithm detected and measured the volume of the cavitary lesion and its proximity to the airway.

A previous algorithm used in small animals^32^ has been improved in performance for human CT scans by using a more accurate lung segmentation algorithm.^33^, ^34^ The validation methods of this higher-resolution algorithm are described in the supplementary methods section of e-Appendix 1. In the case of multiple cavities, we used the cumulative volume of all cavities for analyses.

Fuzzy connectedness methods^35^ were used to segment the airway in high-resolution CT scans (< 4-mm section thickness). The proximity of the cavitary lesion to the airway was determined using Euclidean distance transform.^36^, ^37^, ^38^ If multiple cavities were present, then the cavity closest to the bronchi was used to determine proximity to the airway. To evaluate other radiologic features, a US board-certified radiologist (P. C.) evaluated each scan to indicate presence or absence of consolidation, cavitation, pneumatocele, atelectasis, fibrosis, bronchiectasis, pericardial effusion, pleural effusion, lymphadenopathy, miliary spread, and pneumothorax.

### Statistical Analysis

Data analysis was performed using software (Stata/SE 14.0, Stata Corp). *P* values ≤ .05 were considered statistically significant, and data are shown following recommended numeric presentation. Percentages presented as integers, mean difference (MD) is shown to one decimal place, rate ratio (RR) and hazard ratio (HR) are shown based on the rule of four.^39^, ^40^

Cavitary disease was evaluated based on its volume and proximity to the airway according to data from the computer-automated algorithm. We chose 7 mL as the cutoff between a small and a large cavity and 10 mm as the cutoff between a cavity positioned closer to and farther from the airway to the closest edge of the cavity (inner wall). Cutoff analyses showed significance at these values (*P* < .001 for both) (e-Fig 1). In addition, the presence of bronchiectasis, atelectasis, pleural effusion, and lymphadenopathy were assessed by the radiologist. Other features were too skewed to be compared.

We evaluated baseline cavitary lung disease (cavitary volume and proximity from the cavitary lesion to the airway) with pretreatment cough frequency (negative binomial model) and pretreatment TTP (linear regression), adjusting for age and sex, respectively. We also assessed the association between baseline cavitary lung disease and longitudinal cough frequency results during treatment by using a negative binomial model adjusting for age, culture positivity or negativity, sex, treatment day, and treatment day squared, with a random intercept for study participant; covariates were chosen based on previous analyses.^5^ Baseline cavitary lung disease and longitudinal TTP during treatment were assessed using a linear regression model adjusting for age, cough rate, sex, treatment day, and treatment day squared. A Cox proportional hazards model, unadjusted and adjusted to age and sex, was used to evaluate baseline cavitary lung disease and its effect on cough cessation and culture conversion.

In addition, we used the same analyses to evaluate the presence of baseline atelectasis, bronchiectasis, pleural effusion, and lymphadenopathy on cough frequency before and during treatment, TTP, cough cessation, and culture conversion. For all analyses described, given the small sample size and the exploratory nature of these analyses, no correction for multiple comparisons was made.

### Ethics

This study was conducted in accordance with the Declaration of Helsinki.^41^ This study also was conducted with institutional review board approval by each participating hospital; Universidad Peruana Cayetano Heredia (SIDISI:57183); Asociación Benefica PRISMA in Lima, Peru; and Johns Hopkins University in Baltimore, Maryland (IRB00001676).

## Results

There were 64 participants with available CT scans, but three scans were of poor image quality (recorded as JPEG format instead of Digital Imaging and Communications in Medicine), and two were incomplete (not enough cross-sectional images) and therefore could not be read. After excluding participants with drug-resistant strains, HIV-positive status, or CT scanning performed after 1 month of treatment, 41 participants were available for analysis. The 41 participants in the study group had a total of 695 recordings, but 37% had to be excluded for technical reasons (e-Table 1). After exclusion, there were 18 participants with pretreatment cough recordings. The median length of recordings was 21 hours. Sixty-eight percent of participants were male, with a median age at enrollment of 30 years (interquartile range, 23-50 years). CT scans were obtained a median of 13 days after treatment initiation (interquartile range, 7-21 days). Demographic and radiologic characteristics of participants are shown in Table 1.

**Table 1:** Baseline Demographic Characteristics of the Study Group

Characteristic	Data
No. of participants	41
Male participants, % (95% CI)	28 (68%, 53%-83%)
Age at study enrollment, median (IQR), y	30 (23-50)
Pretreatment culture positive, No. (%, 95% CI)	38 (93%, 84%-100%)
Pretreatment TTP, median (IQR), d	6 (6-8)
Pretreatment negative auramine smear, No. (%, 95% CI)	13 (32%, 17%-47%)
Pretreatment paucibacillary auramine smear,^a^ No. (%, 95% CI)	2 (5%, 0%-12%)
Pretreatment auramine smear,^b^ No. (%, 95% CI)	9 (22%, 9%-35%)
Pretreatment auramine smear,^c^ No. (%, 95% CI)	6 (15%, 3%-26%)
Pretreatment auramine smear,^d^ No. (%, 95% CI)	11 (27%, 13%-41%)
Participants who were drug susceptible No. (%, 95% CI)	41 (100%, 100%-100%)
Lung volume,^e^ median (IQR), mL	4,700 (4,000-6,000)
No cavity,^e^ No. (%, 95% CI)	3 (7%, 0%-16%)
Cavity in right lung only,^e^ No. (%, 95% CI)	16 (39%, 23%-55%)
Cavity in left lung only,^e^ No. (%, 95% CI)	16 (39%, 23%-55%)
Cavity in both lungs,^e^ No. (%, 95% CI)	6 (15%, 3%-26%)
Cavity volume,^e^ median (IQR), mL	4 (1-13)
Distance to airway,^e^ median (IQR), mm	7 (2-16)

IQR = interquartile range; TTP = time to positivity of microscopic observation drug susceptibility culture.

a 1 to 19 acid-fast bacilli per 40 fields at ×400 magnification.

b 20 to 199 acid-fast bacilli per 40 fields at ×400 magnification.

c 5 to 50 acid-fast bacilli per field at ×400 magnification.

d > 50 acid-fast bacilli per field at ×400 magnification.

e Presence, location, and volume of a cavitary lesion are based on computer-automated algorithm results of CT scans, which estimated volumes by using the voxel size. Distance from airway to cavitary lesion was calculated only for participants with a CT scan obtained with a section thickness of 4 mm at most on the basis of the computer-automated algorithm results of CT scans, which estimated distances on the basis of Euclidean distance transform.

According to the radiologist, the most common findings were cavitary lesions (98%), consolidations (93%), bronchiectasis (68%), atelectasis (29%), lymphadenopathy (20%), and pleural effusion (17%). Only three participants had pneumatocele or pericardial effusion reported, only one participant had fibrosis reported, and miliary spread and pneumothorax were not reported.

CT scans with adequate quality were used (n = 41) (Fig 1). In a sensitivity-specificity analysis of cavitation detection, the computer-automated algorithm had a sensitivity of 95% and a specificity of 100% (e-Table 2). The validation of the higher-resolution computer-automated algorithm is shown in the supplementary results (e-Appendix 1, e-Fig 2).

### Cough Frequency Associations

Baseline cavitary volume and proximity to the airway were not associated with pretreatment cough frequency (e-Tables 3-5). However, results of our multivariable analyses showed that cough frequency during treatment in participants with larger cavities was nearly double that of participants with smaller cavities RR, 1.98; 95% CI, 1.17-3.35; *P* = .01) (Table 2). Similarly, participants with cavity lesions located farther from the airway had significantly less cough frequency during treatment than did patients with closer proximities (RR, 0.41; 95% CI, 0.248-0.68; *P* = .001) (Table 3). When we analyzed both cavity volume and distance to the airway, combined, we found that only distance to the airway was significant during treatment (RR, 0.376; 95% CI, 0.196-0.72; *P* = .003) (Table 4). Older age had a strong trend for more cough frequency during treatment in our models (Table 2, Table 3, Table 4).

**Table 2:** Cavity Volume as Risk Factor for Cough Frequency During Treatment

Risk Factor for Cough Frequency	Partially Adjusted Model (N = 41, Obs = 428)			Fully Adjusted Model (N = 41, Obs = 188)		
	RR	*P* Value	95% CI	RR	*P* Value	95% CI
Treatment day	0.90	< .001	0.88-0.93	0.95	< .001	0.92-0.98
Treatment day squared	1.00	< .001	1.00-1.00	1.00	< .001	1.00-1.00
MODS culture positive	…	…	…	1.55	.08	0.94-2.54
Small vs large cavity (categorical)						
Small cavity (≤ 7 mL)	…	…	…	…	…	…
Large cavity (> 7 mL)	1.90	< .001	1.35-2.69	1.98	.01	1.17-3.35
Sex, female	…	…	…	1.31	.3	0.78-2.19
Age per 10 years, y	…	…	…	1.23	.008	1.05-1.42

Cough frequency was used as an outcome in a negative binomial regression to test for risk factors that would increase cough frequency during treatment. In the partially adjusted model, we adjusted for treatment day and treatment day squared. In the fully adjusted model, we adjusted for treatment day, treatment day squared, MODS culture positivity, age, and sex. The volume of the cavity in milliliters was calculated through a computer-automated algorithm that analyzed CT scans on the basis of the voxel size of the cavitary lesion. Participants with no cavities were included in this analysis as having 0-mL volume. MODS = microscopic observation drug susceptibility; Obs = observations; RR = rate ratio.

**Table 3:** Distance to Airway as a Risk Factor for Cough Frequency During Treatment

Risk Factor for Cough Frequency	Partially Adjusted Model (N = 33, Obs = 353)			Fully Adjusted Model (N = 33, Obs = 154)		
	RR	*P* Value	95% CI	RR	*P* Value	95% CI
Treatment day	0.91	< .001	0.89-0.93	0.95	.001	0.92-0.98
Treatment day squared	1.00	< .001	1.00-1.00	1.00	.003	1.00-1.00
MODS culture positive	…	…	…	1.47	.1	0.88-2.48
Distance to airway (categorical)						
Closer distance (≤ 10 mm)	…	…	…	…	…	…
Farther distance (> 10 mm)	0.331	< .001	0.236-0.47	0.41	.001	0.248-0.68
Sex, female	…	…	…	0.92	.8	0.55-1.57
Age per 10 years, y	…	…	…	1.20	.06	1.00-1.46

Cough frequency was used as an outcome in a negative binomial regression to test for risk factors that would increase cough frequency during treatment. In the partially adjusted model, we adjusted for treatment day and treatment day squared. In the fully adjusted model, we adjusted for treatment day, treatment day squared, MODS culture positivity, age, and sex. Distance to the airway from the cavitary lesion was calculated through a computer-automated algorithm that analyzed CT scans with high resolution (< 4-mm section thickness) on the basis of Euclidean distance transform. See Table 2 legend for expansion of abbreviations.

**Table 4:** Combined Risk Factors for Cough Frequency During Treatment

Risk Factor for Cough Frequency	Partially Adjusted Model (N = 33, Obs = 353)			Fully Adjusted Model (N = 33, Obs = 154)		
	RR	*P* Value	95% CI	RR	*P* Value	95% CI
Treatment day	0.91	< .001	0.89-0.93	0.95	< .001	0.91-0.98
Treatment day squared	1.00	< .001	1.00-1.00	1.00	.003	1.00-1.00
MODS culture positive	…	…	…	1.47	.1	0.88-2.46
Small vs large cavity (categorical)						
Small cavity (≤ 7 mL)	Ref	…	…	Ref	…	…
Large cavity (> 7 mL)	1.03	.9	0.68-1.55	0.86	.7	0.42-1.76
Distance to airway (categorical)						
Closer distance (≤ 10 mm)	Ref	…	…	Ref	…	…
Farther distance (> 10 mm)	0.336	< .001	0.227-0.50	0.376	.003	0.196-0.72
Sex, female	…	…	…	0.87	.6	0.49-1.56
Age per 10 years, y	…	…	…	1.20	.06	0.99-1.45

Cough frequency was used as an outcome in a negative binomial regression to test for risk factors that would increase cough frequency during treatment. In the partially adjusted model, we adjusted for treatment day and treatment day squared. In the fully adjusted model, we adjusted for treatment day, treatment day squared, MODS culture positivity, age, and sex. The volume of the cavity in milliliters was calculated through a computer-automated algorithm that analyzed CT scans on the basis of the voxel size of the cavitary lesion. Participants with no cavities were included in this analysis as having 0-mL volume. Distance to the airway from the cavitary lesion was calculated through a computer-automated algorithm that analyzed CT scans with high resolution (< 4-mm section thickness) on the basis of Euclidean distance transform. Ref = reference. See Table 2 legend for expansion of other abbreviations.

There was a nonsignificant trend for association between atelectasis and higher pretreatment cough frequency (RR, 2.71; 95% CI, 0.91-8.1; *P* = .07). Atelectasis (RR, 1.89; 95% CI, 1.17-3.08; *P* = .01) and pleural effusion (RR, 1.99; 95% CI, 1.06-3.73; *P* = .03) were associated with higher cough frequency during treatment (e-Table 6).

### Bacillary Burden Associations

Pretreatment TTP with faster-growing cultures, denoting higher bacillary burden, was associated with larger cavity volumes, in a nonsignificant trend (MD, −1.3; 95% CI, −3.0 to 0.4; *P* = .1) (e-Table 7), but there was no clear trend with proximity to the airway (e-Table 8). However, when analyzing both, combined, farther distance to the airway showed a nonsignificant trend with slower growing cultures, denoting lower bacillary burden (MD, 1.6; 95% CI, −0.6 to 3.9; *P* = .1) (e-Table 9). During treatment, we noted an association between larger cavity volumes and faster culture growth, higher bacillary burden, in sputum (MD, −2.4; 95% CI, −4.6 to −0.3; *P* = .03). Farther distance also was associated with longer time for culture growth, lower bacillary burden, during treatment (MD, 3.3; 95% CI, 1.4-5.2; *P* = .001). When analyzing both volume and distance, combined, only distance to the airway remained significant during treatment (MD, 2.8; 95% CI, 1.0-4.5; *P* = .002). Other radiologic features were not associated with bacillary burden before or during treatment.

### Cough Cessation Assessment

**Figure 2:**
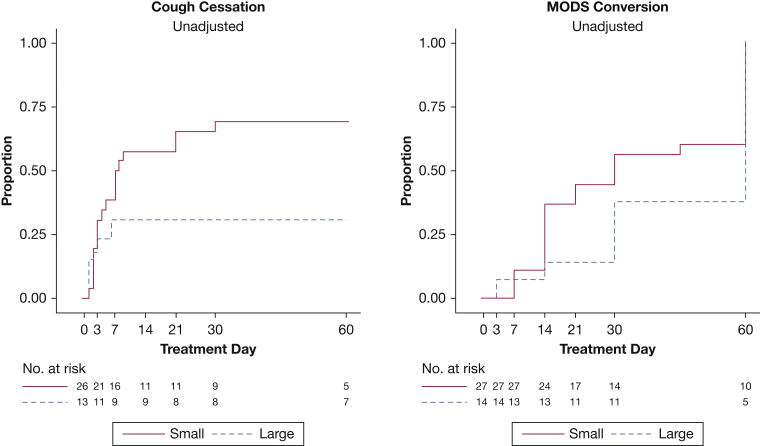
Kaplan-Meier curves for cough cessation and culture conversion by cavity volume size in the study group. Survival curves for cough cessation and microscopic observation drug susceptibility (MODS) culture conversion. Cough cessation represents the time to a cough frequency of ≤ 0.7 cough per hour (considered no cough) for two consecutive recordings. A small cavity is ≤ 7 mL, and a large cavity is > 7 mL on the basis of the results from a computer-automated algorithm. By day 14, the unadjusted probability of cough cessation for small cavities was 58% (95% CI, 40%-77%; adjusted, 97%), whereas for larger cavities this probability was 31% (95% CI, 13%-63%; adjusted, 4%); by day 60, these probabilities were 69% (95% CI, 52%-85%; adjusted, 99%) and 31% (95% CI, 13%-63%; adjusted, 4%), respectively. MODS culture conversion represents time to the first negative culture with no subsequent positive culture. By day 14, the unadjusted probability of culture conversion for small cavities was 37% (95% CI, 22%-58%; adjusted, 32%), whereas for larger cavities this probability was 14% (95% CI, 4%-46%; adjusted, 6%); by day 60, these probabilities were 100% (95% CI, 100%-100%; adjusted, 100%) and 73% (95% CI, 47%-93%; adjusted, 82%), respectively.

**Figure 3:**
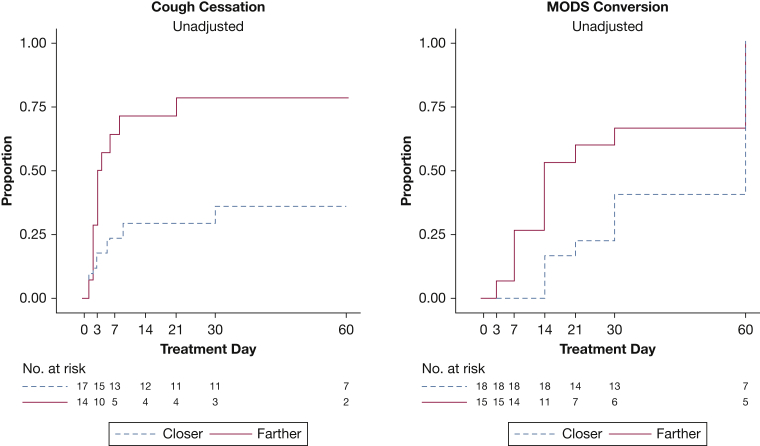
Kaplan-Meier curves for cough cessation and culture conversion by distance from cavity to airway in the study group. Cough cessation represents the time to a cough frequency of ≤ 0.7 cough per hour (considered no cough) for two consecutive recordings. A closer distance is *≤* 10 mm, and a farther distance is > 10 mm on the basis of the results from a computer-automated algorithm. By day 14, the probability of cough cessation for closer distances was 32% (95% CI, 16%-57%; adjusted, 11%), whereas for farther distances this probability was 65% (95% CI, 45%-84%; adjusted, 94%); by day 60, these probabilities were 37% (95% CI, 20%-63%; adjusted, 13.1%) and 75% (95% CI, 55%-91%; adjusted, 98%), respectively. MODS culture conversion represents time to the first negative culture with no subsequent positive culture. By day 14, the probability of culture conversion for closer distances was 15% (95% CI, 5%-40%; adjusted, 2%), whereas for farther distances this probability was 43% (95% CI, 25%-66%; adjusted, 42%); by day 60, these probabilities were 83% (95% CI, 62%-96%; adjusted, 47%) and 100% (95% CI, 100%-100%; adjusted, 100%), respectively. See Figure 2 legend for expansion of abbreviation.

Cough cessation tended to be three times faster among participants with smaller cavities than among those with larger cavities, but this finding was not statistically significant (adjusted HR, 2.89; 95% CI, 0.95-8.8; *P* = .06). The probabilities of cough cessation by day 60, were 69% for small cavities and 31% for large cavities (Fig 2). Furthermore, the hazard for cough cessation was significantly three times higher among participants with cavities located > 10 mm from the airway than among those with cavities located ≤ 10 mm from the airway (adjusted HR, 3.61; 95% CI, 1.26-10.4; *P* = .02). By day 60, the probabilities for cough cessation were 37% for closer distances and 75% for farther distances (Fig 3). The presence of other radiologic features was not associated with cough cessation.

### Culture Conversion Assessment

Culture conversion hazard tended to be two times higher among patients with smaller cavities than among those with larger cavities, but this finding was not statistically significant (adjusted HR, 2.07; 95% CI, 0.90-4.7; *P* = .09). By day 60, the probabilities of culture conversion are 100% for small cavities and 73% for large cavities (Fig 2). Similarly, those with lesions located farther from the airway tended to have a higher culture conversion hazard but this was not statistically significant (adjusted HR, 2.00; 95% CI, 0.95-4.2; *P* = .07). Culture conversion probabilities, by day 60, were 83% for closer distances and 100% for farther distances (Fig 3). The presence of other radiologic features was not associated with culture conversion.

## Discussion

Despite the importance of cough in TB transmission, there is a lack of research on this topic,^2^, ^7^ and a recent clinical guideline demonstrated that cough duration and cavitary lung disease have not been studied.^6^ An increase in cough frequency, as well as delayed cough cessation, heightens the theoretical chances for that patient to expel TB aerosols into the air,^42^, ^43^ increasing the risk of transmission.^44^, ^45^ Our study demonstrated that higher cough frequency during treatment, as well as delayed time to cough cessation, are associated with larger cavitary volume, especially cavities closer to the airway.

Patients suspected of having pulmonary TB possibly could be risk stratified for transmission and prognosis within 24 hours through use of the CayeCoM and chest CT scan by using a diagnostic algorithm, based on an underlying mathematical framework,^46^ in a much shorter time frame compared with that for culture (median culture of MODS is 1 week).^47^ This risk stratification is particularly important in TB, for which transmission is heterogeneous,^9^, ^10^, ^11^, ^12^, ^13^, ^14^, ^15^, ^16^, ^17^, ^18^, ^19^ especially in certain environments.^48^ A diagnostic algorithm could determine quickly the most likely contagious patients, as well as identify potential patients who might not respond well to treatment given their increased disease burden.^46^ However, the most important factor to diminish transmission is effective treatment,^5^, ^49^, ^50^ and other factors (cough strength, sputum viscosity, cough hygiene, social interaction) also would need to be evaluated for this algorithm to build on current scores.^51^

We observed that a larger cavity volume and a closer proximity to the airway was associated with higher cough frequency during treatment, higher bacillary burden before and during treatment, delayed cough cessation, and culture conversion. Previous studies support the association between larger cavitary volume and higher bacillary burden before treatment,^52^ as well as a relationship between closer proximity to the airway and higher bacillary burden before treatment.^53^ When evaluating both volume and proximity, combined, we found that of these two, proximity to the airway seems to play a larger role for both cough frequency during treatment and bacillary burden before and during treatment. The closer the cavity is to the airway, the more inflammation causes increased cough frequency during treatment, and the better oxygen access is provide an optimum microenvironment for *Mycobacterium tuberculosis* growth.^54^, ^55^ Previous studies show that *M tuberculosis* grows better within the macrophages of the luminal surface of the cavitary lesion because of better oxygen access, coupled with a lack of T lymphocytes, which diminishes the interactions between T cells and macrophages that clear mycobacteria.^54^, ^55^

Patients with more severe infection might have bronchial obstruction that can lead to a lung collapse (atelectasis), which in turn can act as a one-way valve that ultimately increases cough frequency.^56^, ^57^ However, pleural effusion is a hypersensitivity reaction that could cause a systemic response resulting in cough, independent of bacillary burden.^58^, ^59^, ^60^ Our study also supports the suggested relationship between radiologic extent of the disease, based on CXR,^61^ and cough frequency.^62^

A limitation is that 18 participants had pretreatment recordings, and nearly one-third of recordings had to be excluded due to technical limitations. We did not identify bias when comparing participants with at least 10 excluded recordings with those with fewer than 10 excluded recordings. Chest radiography (CXR) is usually the imaging modality of choice in TB control programs, but CT scans are more sensitive for detecting pleural and parenchymal lesions.^63^, ^64^, ^65^, ^66^, ^67^, ^68^ Nearly all participants had cavities, so we could not evaluate or compare the cough frequency between patients with a cavity and patients without a cavity. The presence of a cavity has not been associated with cough-generated aerosols.^49^ Given that CXR are obtained in a two-dimensional fashion, it would not have enabled us to evaluate three-dimensional volume and proximity to the airway. A strength of our investigation is that our cough measurements with CayeCoM were validated previously,^69^, ^70^, ^71^ as was the algorithm used to evaluate cavity volume and proximity to the airway.^32^, ^38^ Our sample size was similar to those in other CT scanning studies in TB, and the small delay in CT scanning after starting treatment is unlikely to affect results because major changes in cavity structure are uncommon in the first month of treatment.^28^, ^52^, ^53^, ^64^

## Conclusions

To our knowledge, this is the first report regarding an association between cough frequency during treatment and cavitary lung disease. Our study demonstrates an association between cough frequency during treatment, and its duration, with cavitary volume and cavitary proximity to the airway. Younger patients, with small cavitary lesions, especially lesions farther from the airway, may present with minimal cough and sputum samples with low bacillary burden (ie, be smear negative). These patients likely would have cough symptoms later than those with cavities close to the bronchi and, if they are not cultured, may be missed by smear alone. Similarly, if a patient is has a large cavity diagnosed, especially close to the airway, this patient has an increased risk for coughing more during treatment and should be monitored closely for the possibility of expelling more *M tuberculosis* to the environment.

## Supplementary Data

e-Online Data
