# Supplementary material for: Cough Frequency During Treatment Associated With Baseline Cavitary Volume and Proximity to the Airway in Pulmonary TB
Source: Chest. 2018 Mar 17;153(6):1358–67. doi: 10.1016/j.chest.2018.03.006 (PMC6026292; doi:10.1016/j.chest.2018.03.006)
Supplement: e-Online Data [file mmc1.pdf]

CHEST Online Supplement

Cough Frequency During Treatment Associated With Baseline Cavitary Volume and Proximity to the Airway in Pulmonary TB

Alvaro Proaño, MD; David P. Bui, MPH; José W. López, MD; Nancy M. Vu, MD; Marjory A. Bravard, MD; Gwenyth O. Lee, PhD; Brian H. Tracey, PhD; Ziyue Xu, PhD; Germán Comina, PhD; Eduardo Ticona, PhD; Daniel J. Mollura, MD; Jon S. Friedland, PhD; David A. J. Moore, MD; Carlton A. Evans, PhD; Philip Caligiuri, MD; Robert H. Gilman, MD; for the Tuberculosis Working Group in Peru

CHEST 2018; 153(6):1358-1367

Online supplements are not copyedited prior to posting and the author(s) take full responsibility for the accuracy of all data.

© 2018 AMERICAN COLLEGE OF CHEST PHYSICIANS. Reproduction of this article is prohibited without written permission from the American College of Chest Physicians. See online for more details. **DOI:** 10.1016/j.chest.2018.03.006

e-Appendix 1

Supplementary Methods

Algorithm Validation

Based on bi-dimensional reporting by the study radiologist, we calculated the estimated volume for each cavity. The study radiologist evaluated the maximum diameter measurements (x) in the axial (transverse) plane and its perpendicular diameter (y). Measurements are maximum diameters from outer wall to outer wall. We then calculated the radiologist estimated-volume by using a volumetric approximation for an ellipsoid based on these two measurements (x, y), as follows: $\frac{4}{3}\pi(\frac{x}{2})^2\frac{y}{2}$, similar to what has been used to estimate lung cancer volume.[1] Each radiologist-estimated volume was compared to the cavity volume obtained from the computer-automated algorithm. Cavity volumes were calculated based on their voxel size, representing the three-dimensional size of the cavity, the wall was not included in this measurement. [2-4] To reduce potential error of measuring multiple cavities due to image crowding, Spearman's correlations were calculated between cavity volumes only for films that only had a single cavity found by both the radiologist and the algorithm. Secondly, Spearman's correlations were calculated between all films (cumulative volumes). Additionally, we evaluated the relationship between cavity volume and distance to the airway, as measured by the computer-automated algorithm.

Supplementary Results

Algorithm Validation

There was a strong correlation between the radiologist-estimated cavity volume and the cavity volume from the computer-automated algorithm, for both films with single cavity lesions (n=14, Rho=0.90, p<0.001) and in all films (n=41, Rho=0.78, p<0.001). The correlations between cumulative radiologist-estimated volumes and cumulative algorithm-estimated volumes are shown in **e-Figure 2**. The mean cumulative radiologist-estimated volume size was 37.42-mL (standard deviation[SD]=10.06-mL) and the computer-automated algorithm estimated volume size was 16.19-mL (SD=5.73-mL).

Additionally, based on the computer-automated algorithm results, we found that cavity volume and distance to the airway are correlated (Rho=-0.61, p<0.001), where the larger the volume the closer the cavity is to the airway.

Online supplements are not copyedited prior to posting and the author(s) take full responsibility for the accuracy of all data.

References

1. Hayes SA, Pietanza MC, O'Driscoll D, et al. Comparison of CT volumetric measurement with RECIST response in patients with lung cancer. Eur J Radiol. 2016;85(3):524-533.

2. Xu Z, Bagci U, Kubler A, et al. Computer-aided detection and quantification of cavitary tuberculosis from CT scans. Med Phys. 2013;40(11):113701.

3. Mansoor A, Bagci U, Xu Z, et al. A generic approach to pathological lung segmentation. IEEE Trans Med Imaging. 2014;33(12):2293-2310.

4. Proaño A, Xu Z, Caligiuri P, Mollura DJ, Gilman RH, Tuberculosis Working Group in Peru. Computer automated algorithm to evaluate cavitary lesions in adults with pulmonary tuberculosis. J Thorac Dis. 2017;9(1):E93-E96.

Online supplements are not copyedited prior to posting and the author(s) take full responsibility for the accuracy of all data.

e-Figure 1 – Frequency Distribution Analysis for Cavity Volumes and Distances

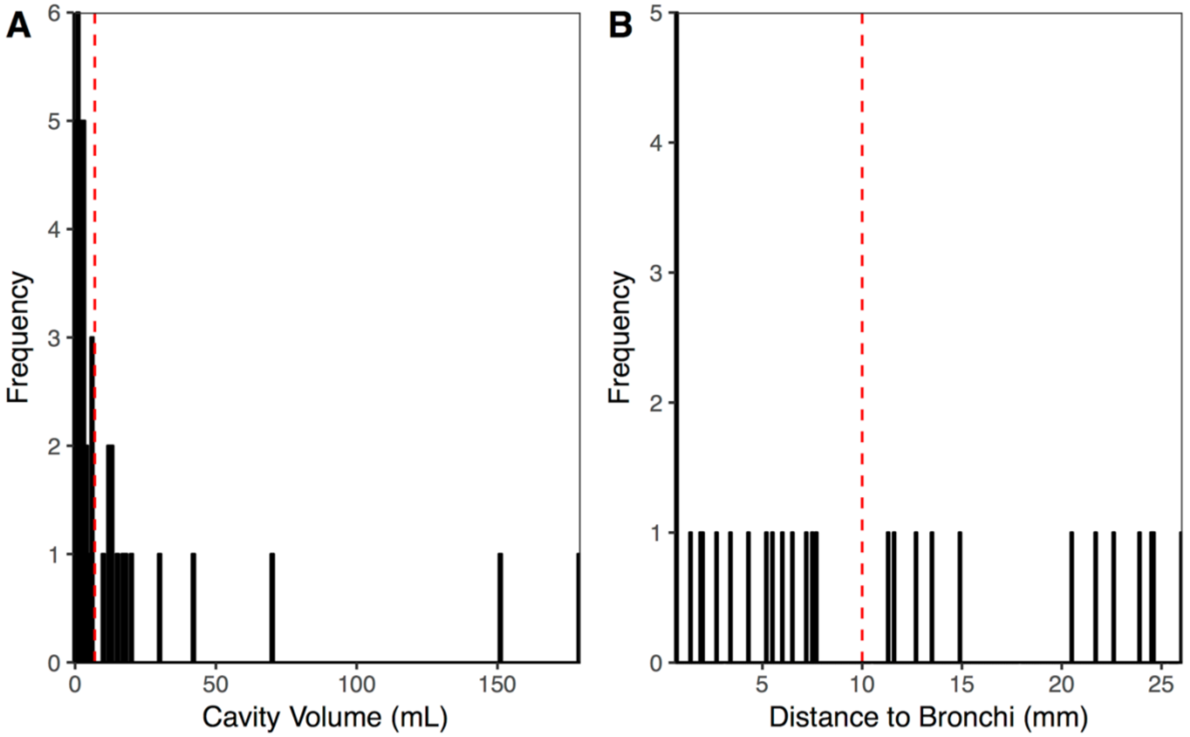

e-Figure 1(A) shows a histogram distribution, using a bin-width of 1.0-mL, of cavity volumes in all 41 CT scans. Results from a computer-automated algorithm. Participants with no cavities are recorded as having 0.0-mL volume. There is a cutoff at 7-mL, which is depicted by the red line. The median cavity volume for the subgroup less than or equal to 7-mL is of 1.89-mL, and the median cavity volume for the subgroup greater than 7-mL is of 17.40-mL. When comparing both subgroups, by Kruskal-Wallis rank sum test, there is a significant statistical difference ($p<0.001$). **e-Figure 1(B)** shows a similar histogram distribution, using a binwidth of 0.1-mm, of the distance between the cavity and the airway, only high-resolution CT scans were used (n=30) for this analysis, based on computer-automated algorithm, which also shows a clear cutoff at 10-mm. The median distance to bronchi for the subgroup less than or equal to 10-mm is of 3.04-mm, and the median distance to bronchi for the subgroup greater than 10-mm is of 21.13-mL. When comparing both subgroups, by Kruskal-Wallis rank sum test, there is a significant statistical difference ($p<0.001$).

Online supplements are not copyedited prior to posting and the author(s) take full responsibility for the accuracy of all data.

e-Figure 2 – Correlation Between Radiologist –Estimated Cavity Volume and Algorithm-Estimated Cavity Volume

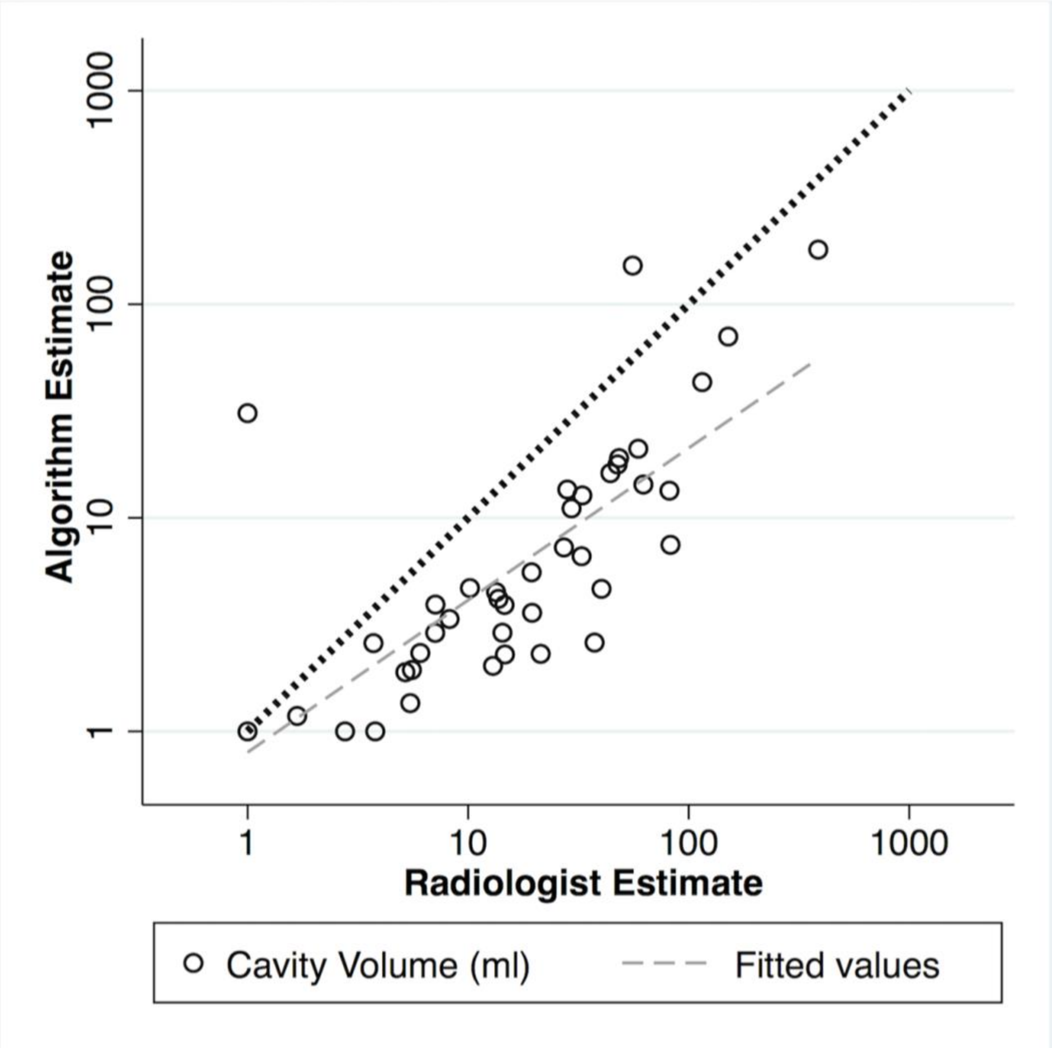

Spearman correlation evaluating the reporting of cavity volumes by the study radiologist vs. computer-automated algorithm, on a log scale (n=41). The correlations were high, with a Spearman rho of 0.78 ($p<0.001$). The radiologist-estimated volume is calculated based on the bi-dimensional readings of the radiologist, where the radiologist identified the maximum diameter measurement on the axial (transverse plane), defined as x, and y is defined as its perpendicular diameter. Measurements are maximum diameters from outer wall to outer wall. We used a volumetric approximation for an ellipsoid based on these two measurements (x, y), as follows: $\frac{4}{3}\pi(\frac{x}{2})^2\frac{y}{2}$. Both x and y have to be divided by 2 to obtain the radii, respectively. Cavity volumes based on the computer-automated algorithm were calculated based on their voxel size, representing the three-dimensional size of the cavity, the wall was not included in this measurement. The dotted line represents equality (x=y).

Online supplements are not copyedited prior to posting and the author(s) take full responsibility for the accuracy of all data.

e-Table 1 – Cough Recordings in Study Group

	Study Group
**Number of participants evaluated**	41
**Total recordings**	695
**Recordings excluded from analysis (%)**	255 (37%)
… recording malfunction	17
… recordings with high background noise	174
… MP3 to WAV conversion error	5
… recordings not checked by nurse	23
… recordings started after 24 hours	10
… recordings shorter than 1 hour	26
**Recordings contributing to analysis**	440 (432 total days)
**Hours contributing to analysis**	8,089

Recordings were obtained with the Cayetano Cough Monitor (CayeCoM) which evaluated participants' cough pre-treatment and during treatment for their pulmonary tuberculosis. All participants were HIV-negative, culture-confirmed tuberculosis, TB drug-susceptible to isoniazid and rifampicin, and had an adequate CT scan obtained within a month of treatment initiation. The median length of recordings was of 21 hours; recordings started at 09:00 AM. There were 10 participants who had at least 10 of their cough recordings excluded and we found no significant differences between them and the 31 participants with less than 10 recordings excluded in age, sex, cavity volume, proximity to the bronchial tree, cough frequency in their first appropriate recording.

Online supplements are not copyedited prior to posting and the author(s) take full responsibility for the accuracy of all data.

e-Table 2 – Sensitivity/Specificity Analysis based on Cavity Detection

Computer-automated algorithm detected any cavity?	U.S. board-certified radiologist detected any cavity?		
	**Yes**	**No**	**Total**
**Yes**	38	0	38
**No**	2	1	3
**Total**	40	1	41

A U.S. board-certified evaluated CT scans to determine if there was presence of a cavity or not (gold standard). A computer-automated algorithm was assessed against the results from the radiologist, showing sensitivity 95% (95% CI=83% – 99%) and specificity of 100% (95% CI=3% – 100%).

Online supplements are not copyedited prior to posting and the author(s) take full responsibility for the accuracy of all data.

e-Table 3 – Cavity Volume and Pre-Treatment Cough Frequency

	**Adjusted Model N=18, Obs=18**			
	**RR**	**p**	**95% CI**	
**Small vs Large Cavity (Categorical)**				
Small Cavity (≤ 7-mL)	*ref*			
Large Cavity (> 7-mL)	0.59	0.4	0.173	2.02
**Sex, Female**	0.65	0.5	0.186	2.28
**Age, Years (per 10 y)**	1.08	0.8	0.66	1.77

The participants with pre-treatment cough recordings were evaluated against their cavity volume adjusting for sex and age using a negative binomial regression model. Cavity volumes based on the computer-automated algorithm were calculated based on their voxel size, representing the three-dimensional size of the cavity, the wall was not included in this measurement. Ratios presented based on the rule of four. CI = Confidence Interval; mL = Milliliters; Obs = Observations; RR = Rate Ratio; y = Years.

Online supplements are not copyedited prior to posting and the author(s) take full responsibility for the accuracy of all data.

e-Table 4 – Distance to the Airway and Pre-Treatment Cough Frequency

	Adjusted Model N=17, Obs=21			
	**RR**	**p**	**95% CI**	
**Distance to Airway (Categorical)**				
Closer distance (≤10-mm)	*ref*			
Farther distance (>10-mm)	0.57	0.2	0.228	1.44
**Sex, Female**	1.08	0.8	0.52	2.21
**Age, Years (per 10 y)**	0.94	0.7	0.65	1.34

The participants with pre-treatment cough recordings were evaluated against distance to the airway adjusting for sex and age using a negative binomial regression model with random effects. Distance to the airway from the cavity was calculated through a computer-automated algorithm that analyzed computerized tomography scans with high resolution (< 4-mm slice thickness), based on Euclidean distance transform. Ratios presented based on the rule of four. CI = Confidence Interval; mm = Millimeters; Obs = Observations; RR = Rate Ratio; y = Years.

Online supplements are not copyedited prior to posting and the author(s) take full responsibility for the accuracy of all data.

e-Table 5 – Cavity Volume, Distance to the Airway and Pre-Treatment Cough Frequency

	Adjusted Model N=17			
	**RR**	**p**	**95% CI**	
**Small vs Large Cavity (Categorical)**				
Small Cavity (≤ 7-mL)	*ref*			
Large Cavity (> 7-mL)	0.45	0.3	0.010	1.99
**Distance to Airway (Categorical)**				
Closer distance (≤10-mm)	*ref*			
Farther distance (>10-mm)	0.64	0.6	0.096	4.3
**Sex, Female**	0.48	0.4	0.101	2.27
**Age, Years (per 10 y)**	1.02	0.9	0.59	1.76

The participants with pre-treatment cough recordings were evaluated against their cavity volume and distance to the airway, in a combined model, adjusting for sex and age using a negative binomial regression model. Cavity volumes based on the computer-automated algorithm were calculated based on their voxel size, representing the three-dimensional size of the cavity, the wall was not included in this measurement. Distance to the airway from the cavity was calculated through a computer-automated algorithm that analyzed computerized tomography scans with high resolution (< 4-mm slice thickness), based on Euclidean distance transform. Ratios presented based on the rule of four. CI = Confidence Interval; mL = Milliliters; mm = Millimeters; RR = Rate Ratio; y = Years.

Online supplements are not copyedited prior to posting and the author(s) take full responsibility for the accuracy of all data.

e-Table 6 – Risk Factors for Cough Frequency During Treatment Based on Radiological Features

	Partially Adjusted Model N=41, Obs=428				Fully Adjusted Model N=41, Obs=188			
	RR	p	95% CI		RR	p	95% CI	
**Atelectasis**	1.17	0.3	0.83	1.64	1.89	0.01	1.17	3.08
**Bronchiectasis**	1.44	0.04	1.01	2.04	1.17	0.5	0.70	1.96
**Pleural Effusion**	2.47	<0.001	1.68	3.66	1.99	0.03	1.06	3.73
**Lymphadenopathy**	0.60	0.008	0.41	0.87	0.83	0.5	0.46	1.49

Negative binomial regression models adjusting for treatment day and treatment day squared in the partially adjusted model and for age, MODS culture positivity, sex, treatment day, and treatment day squared, with a random intercept for study participant (N=41 for all models) in the fully adjusted model. Assessing four different radiological features (atelectasis, bronchiectasis, pleural effusion, and lymphadenopathy). Based on readings from U.S. board-certified radiologist. Consolidations were not assessed because the majority of participants (38/41) had this feature. Cavitations were not assessed because almost all participants (40/41) had at least one cavitation. Pneumatocele, fibrosis, pericardial effusion, miliary spread and pneumothorax were not analyzed due to small reporting of these features. Ratios presented based on the rule of four. CI = Confidence Interval; Obs = Observations; RR = Rate Ratio; y = Years.

Online supplements are not copyedited prior to posting and the author(s) take full responsibility for the accuracy of all data.

e-Table 7 – Cavity Volume and Pre-Treatment Time to Positivity of Cultures

	Adjusted Model N=38			
	**MD**	**p**	**95% CI**	
**Small vs Large Cavity (Categorical)**				
Small Cavity (≤ 7-mL)	*ref*			
Large Cavity (> 7-mL)	-1.3	0.1	-3.0	0.4
**Sex, Female**	1.3	0.1	-0.4	3.1
**Age, Years (per 10 y)**	-0.2	0.6	-0.7	0.4

Using a linear regression model to evaluate the association between cavity volume and pre-treatment time to positivity of positive culture samples using the microscopic-observation drug susceptibility (MODS) broth culture. Cavity volumes based on the computer-automated algorithm were calculated based on their voxel size, representing the three-dimensional size of the cavity, the wall was not included in this measurement. Numbers presented to one decimal place. CI = Confidence Interval; MD = Mean Difference; mL = Milliliters; Ref = Reference; y = Years.

Online supplements are not copyedited prior to posting and the author(s) take full responsibility for the accuracy of all data.

e-Table 8 – Distance to the Airway and Pre-Treatment Time to Positivity of Cultures

	Adjusted Model N=30			
	**MD**	**p**	**95% CI**	
**Distance to Airway (Categorical)**				
Closer distance (≤10-mm)	*ref*			
Farther distance (>10-mm)	2.0	0.5	0.03	3.9
**Sex, Female**	1.7	0.1	-0.4	3.7
**Age, Years (per 10 y)**	-0.3	0.3	-1.0	0.3

Using a linear regression model to evaluate the association between distance of the cavity to the airway and pre-treatment time to positivity of positive culture samples using the microscopic-observation drug susceptibility (MODS) broth culture. Distance to the airway from the cavity was calculated through a computer-automated algorithm that analyzed computerized tomography scans with high resolution (< 4-mm slice thickness), based on Euclidean distance transform. Numbers presented to one decimal place, except the lower bound of the confidence interval in farther distance to have at least one significant digit. CI = Confidence Interval; MD = Mean Difference; mm = Millimeters; Ref = Reference; y = Years.

Online supplements are not copyedited prior to posting and the author(s) take full responsibility for the accuracy of all data.

e-Table 9 – Cavity Volume, Distance to the Airway and Pre-Treatment Time to Positivity of Cultures

	Adjusted Model N=30			
	**MD**	**p**	**95% CI**	
**Small vs Large Cavity (Categorical)**				
Small Cavity (≤ 7-mL)	*ref*			
Large Cavity (> 7-mL)	-0.8	0.5	-3.1	1.5
**Distance to Airway (Categorical)**				
Closer distance (≤10-mm)	*ref*			
Farther distance (>10-mm)	1.6	0.1	-0.6	3.9
**Sex, Female**	1.5	0.2	-0.7	3.6
**Age, Years (per 10 y)**	-0.3	0.3	-1.0	0.3

Using a linear regression model to evaluate the association between cavity volume and distance to the airway, in a combined model, and pre-treatment time to positivity of positive culture samples using the microscopic-observation drug susceptibility (MODS) broth culture. Cavity volumes based on the computer-automated algorithm were calculated based on their voxel size, representing the three-dimensional size of the cavity, the wall was not included in this measurement. Distance to the airway from the cavity was calculated through a computer-automated algorithm that analyzed computerized tomography scans with high resolution (< 4-mm slice thickness), based on Euclidean distance transform. Numbers presented to one decimal place. CI = Confidence Interval; MD = Mean Difference; mL = Milliliters; mm = Millimeters; Ref = Reference; y = Years.

Online supplements are not copyedited prior to posting and the author(s) take full responsibility for the accuracy of all data.
